# Quiescent and Active Tear Protein Profiles to Predict Vernal Keratoconjunctivitis Reactivation

**DOI:** 10.1155/2016/9672082

**Published:** 2016-02-17

**Authors:** Alessandra Micera, Antonio Di Zazzo, Graziana Esposito, Roberto Sgrulletta, Virginia L. Calder, Stefano Bonini

**Affiliations:** ^1^IRCCS-G.B. Bietti Foundation, 00100 Rome, Italy; ^2^Department of Ophthalmology, Campus Bio-Medico University, 00128 Rome, Italy; ^3^UCL Institute of Ophthalmology, London EC1V 9EL, UK

## Abstract

*Objective*. Vernal keratoconjunctivitis (VKC) is a chronic recurrent bilateral inflammation of the conjunctiva associated with atopy. Several inflammatory and tissue remodeling factors contribute to VKC disease. The aim is to provide a chip-based protein analysis in tears from patients suffering from quiescent or active VKC.* Methods*. This study cohort included 16 consecutive patients with VKC and 10 controls. Participants were subjected to clinical assessment of ocular surface and tear sampling. Total protein quantification, total protein sketch, and protein array (sixty protein candidates) were evaluated.* Results*. An overall increased Fluorescent Intensity expression was observed in VKC arrays. Particularly, IL1*β*, IL15, IL21, Eotaxin2, TACE, MIP1*α*, MIP3*α*, NCAM1, ICAM2, *β*NGF, NT4, BDNF, *β*FGF, SCF, MMP1, and MMP2 were increased in quiescent VKC. Of those candidates, only IL1*β*, IL15, IL21, *β*NGF, SCF, MMP2, Eotaxin2, TACE, MIP1*α*, MIP3*α*, NCAM1, and ICAM2 were increased in both active and quiescent VKC. Finally, NT4, *β*FGF, and MMP1 were highly increased in active VKC.* Conclusion*. A distinct “protein tear-print” characterizes VKC activity, confirming some previously reported factors and highlighting some new candidates common to quiescent and active states. Those candidates expressed in quiescent VKC might be considered as predictive indicators of VKC reactivation and/or exacerbation out-of-season.

## 1. Introduction

Vernal keratoconjunctivitis (VKC) is a multifactorial eye disease associated with atopy, characterized by a chronic recurrent bilateral inflammation of the conjunctiva [1]. This childhood disease resolves spontaneously at puberty, although complications might occur due to severe and/or long-standing inflammation, leading to fibrovascular reaction, new collagen deposition, huge tissue remodeling, and permanent visual changes [2]. Recurrent local inflammation might also trigger corneal impairment as well as undesired corneal ulcers [2]. A late onset VKC-like disease has been also observed in young adults with signs/symptoms resembling the childhood disease and characterized by minor corneal involvement [3]. VKC inflammation is variable (mild, moderate, or severe), ranging from seasonal (acute) to chronic, and resembling the perennial seasonal conjunctivitis [2].

Current knowledge indicates that several inflammatory and tissue remodeling factors contribute to signs and symptoms of VKC [4]. Infiltrating Th2 cells and eosinophils, recruited mast cells and activated fibroblast/myofibroblasts drive the chronic inflammatory process by releasing soluble mediators, cytokines/chemokines, adhesion molecules, neuropeptides, and growth factors [5–9]. Secreted proteins accumulate in the tear fluid, representing a “tear-print” of the inflamed ocular surface and a view to physiopathological status. Increased cytokines belonging to Th1 (IFN*γ*, IL2) and Th2 (IL4, IL5) subgroups have been detected in tears and conjunctival impression cytologies (IC) from VKC patients [7–10]. In addition, chemokines/adhesion molecules, growth/angiogenic/profibrogenic factors, and receptors of innate immunity contribute actively to the local inflammation, as observed in both tears and ICs, or provided by* in vitro* studies [11–13]. Advances in protein analysis have been done to provide new high-throughput technological methods to identify several proteins at once in biological samples, including tears and conjunctival specimens obtained according to the IC technique [14, 15].

To date, different clinical features and therapeutic outcome in VKC suggest the need for predictive approaches, the crosstalk between a new grading approach and suitable biomarkers [15–19]. Therefore, the aim of this study was to provide a comprehensive protein expression profile in tears from active and quiescent VKC, by using a tear-chip array coupled analysis of 60 different factors (herein referred to as candidates). The final attempt will be to identify some candidates (laboratory biomarkers) associated with VKC activity, in order to identify those candidates, of prognostic value, linked to a potential background allowing VKC reactivation.

## 2. Materials and Methods

### 2.1. Ethical Statement and Reagent/Plastic-Ware Information

Authorization to carry out the study was provided by the intramural Ethical Committee at the University Hospital Campus Bio-Medico (Rome, Italy). The approval included patient management, tear sampling, and full experimental procedures. Furthermore, all procedures of handling human samples were conducted in accordance with guidelines established by the Association for Research in Vision and Ophthalmology and adhered to the tenets of the Declaration of Helsinki with respect to human subjects.

Unless specified in the text, sterile plastic-ware and analytical grade reagents were from NUNC (Roskilde, Denmark), SERVA (Heidelberg, Germany), ICN (Costa Mesa, CA), Euroclone (Milan, Italy), and Sigma-Aldrich (St. Louis, MA, USA). Ultrapure MilliQ-Grade water was daily provided (DirectQ5 Millipore, Vimodrone, Italy).

### 2.2. Study Population and Tear Collection

After an accurate explanation of the study design and the description of the potential information arising from the study, the participants (patients and controls or their parents) provided written informed consent to proceed to tear sampling.

A total of 16 consecutive patients suffering from VKC (15 M/1 F; mean age 15.50 ± 3.16 yrs, ranging from 8 to 20 yrs) were included in the study (all referring spontaneously to our Clinical Unit). Enrollment criteria included a positive history of VKC and the absence of topical/systemic therapy. VKC diagnosis was based on ocular surface inflammation characterized by itching, photophobia, tearing/mucous discharge, the presence of a giant papillary reaction on the upper tarsal conjunctiva, and/or at the limbus associated with the presence of eosinophils in conjunctival scrapings [4]. Exclusion criteria included the presence of coexisting ocular and/or ocular surface diseases; the presence of systemic diseases other than coexisting allergic rhinitis, asthma, or atopic dermatitis; use of contact lens; use of topical/systemic medications at the time of the sample collection. Itching, tearing, photophobia, and foreign body sensation symptoms as well as the conjunctival hyperemia, mucous discharge, papillae, and corneal epithelial defects signs were scored from 0 to 3 (0: absent, 1: weak, 2: mild, and 3: severe). Finally, patients were graded according to the following severity score:* 0-quiescent*, absence of ocular symptoms;* 1-mild*, presence of ocular symptoms but not photophobia;* 2-moderate*, presence of symptoms and photophobia;* 3-severe*, presence of ocular symptoms and mild to moderate superficial punctuate keratitis (SPK);* 4-very severe*, presence of diffuse superficial keratopathy and/or corneal ulcer [4].

Ten sex/age matched healthy control volunteers with no signs/symptoms of conjunctivitis or ocular surface disease and not receiving systemic/local medications (steroids and/or antiallergic drugs or eye-drop tear substitute or surgical treatment) were enrolled for appropriate comparisons (5 M/5 F; mean age 13.50 ± 7.56 yrs, ranging from 8 to 29 yrs).

At the end of ophthalmic examination, the subjects underwent nonanesthetized tear collection. Briefly, tears were sampled according to the standardized “eye-flush” procedure implying the addition of 50 *µ*L sterile Balanced Salt Solution (BSS; Alcon Laboratories Inc., Fort Worth, TX) and a quick collection of tears with sterile single-wrapped plastic micropipettes (Sigma) [20–22]. The eye-flush procedure is consistent with the choice to collect tears in an extreme suitable/comfortable way for this “younger and anxious” study population [20, 21]. All tear samples were quickly supplemented with protease inhibitors (Pierce Biotechnology, Rockford, IL) and stored at −20°C. Delivery from Clinical Unit to the Laboratory Unit was performed using an isothermal cage (CryoCooler; Starlab Intl GmbH, Ahrensburg, Germany), avoiding temperature changes, according to national rules and standardized operating procedures.

### 2.3. Protein Extraction, Quantification, and Electrophoresis

Tear samples were diluted in cold lysis buffer (50 mM Tris-HCl, 150 mM NaCl, 1 mM EDTA, 0.1% Nonidet P-40, 1 mM NaF, and 1 mM PMSF; pH 7.5), briefly sonicated (VibraCell; Sonics, Newtown, CT), and clarified by centrifugation (13000 rpm/7 min). For protein quantification, 3 *µ*L samples were analyzed with the A280 program (Nanodrop; Celbio, Milan, Italy). Tear samples showing low protein amounts were from patients/subjects having symptoms of ocular dryness and reduced mucus production. Protein separation was performed under reducing conditions on 4–12% mini-SDS polyacrylamide gels (SDS-PAGE; 150 V/frontline; Miniprotean3 apparatus; Biorad, Hercules, CA, USA) and the bands were transferred to Hybond membranes (GE Healthcare, Buckinghamshire, UK) under semidry conditions (13 V/25 min; semidry transblot system; Biorad). Prestained broad weight marker was run in parallel (10–250 kDa prestained marker; Biorad). Bands were visualized by Ponceau S. Samples having high albumin/IgGs protein profiles were cleared (GE Healthcare).

### 2.4. Chip-Based Protein Arrays

Protein analysis was performed on chip-based arrays provided on glass slides, each comprising 14 identical subarrays containing 60 factors (antibody spots in duplicate) retrospectively selected (literature search) for custom-built array chips (Ray Biotech, Norcross, CA). Briefly, normalized protein extracts (350 ng/mL; 70 *µ*L/well) were diluted and hybridized in subarrays. VKC/control samples were processed simultaneously and the procedures of sample dilution, incubation/washing, detection, and labeling were according to the manufacturer's recommendation. Spin-dried slides were scanned in a GenePix 4400 Microarray platform (Molecular Devices LLC, Sunnyvale, Silicon Valley, CA). The specific area (grid; array/spot) was first manually spotted and then automatically adjusted by the software, and the capturing conditions were routinely applied to all glass slides. Images were uniformly adjusted for size, brightness, contrast, and chip-to-chip comparisons by the software, and provided as 8-bit Tiff converted format (Axon GenePix Pro 6.0 software; Molecular Devices). Interassay normalization was guaranteed by the presence of multiple internal controls for each subarray. The minimum sensitivity range of the array was 3.8–56 pg/mL.

### 2.5. Statistical Analysis

The pooled-samples choice was not considered in this cohort study, guaranteeing good statistical power and biological sensitivity/variance. The Fluorescent Intensity (FI) values (spot) were obtained by subtracting the background signal (GenePix Pro 6.0 software). Single FI values were entered into a Microsoft Excel database (Microsoft, Redmond, WA) and duplicate spots outside the 10% coefficient of variability were refused from the statistical analysis. FDR value of 0.01 was set. FI averages (means ± SD) were automatically calculated from replicates (2 spots) of not-pooled tear samples. Comparisons between VKC and control groups or within VKC groups were performed by using the two-sided unpaired *t*-test analysis (SPSS ver. 15; IBM Inc., Chicago, IL). As cut-offs, ≥ 2-fold changes (FC; herein defining the abundance in a given candidate (factor) with respect to control) and *p* ≤ 0.05 or *p* < 0.00083 (0.05 *p* value/60 targets) for multiple testing with the Bonferroni correction were considered. Correlations between differentially expressed candidates and clinical findings were calculated by Spearman correlation rank test (rho ≥ 0.5 and *p* ≤ 0.05 or *p* < 0.00083). Volcano plots and Venn diagrams were used to illustrate microarray data sets and results [23, 24].

## 3. Results

**Figure 1:**
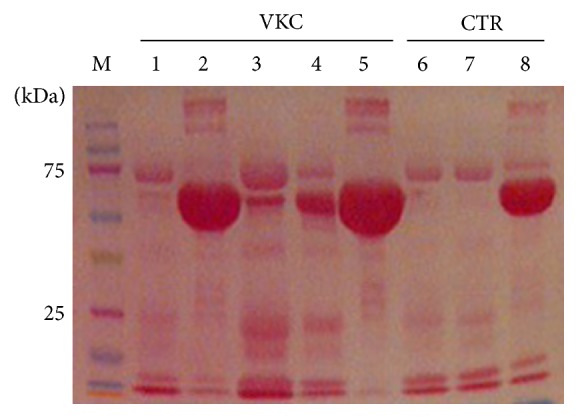
Tear protein profile. Equal protein amounts (20 *µ*g/lane/sample) were subjected to electrophoretic separation (SDS-PAGE) and membranes were stained with Ponceau S before image acquisition (see M&M). Note the presence of albumin (60 kDa), low/high IgG bands (40/100 kDa), and fibronectin (200 kDa) in some tear samples (1–5, VKC; 6–8 healthy controls). To retrieve low-expressed antigens, samples showing high albumin/IgGs were treated with a specific preclearing kit (see M&M).

The entire study population included 16 consecutive VKC patients and 10 normal subjects (see M&M). The clinical and biochemical data of VKC patient group and subgroups are summarized in Table 1. Briefly, nine out of 16 patients referred to our Clinical Unit out-of-season, having no symptoms of disease (*quiescent VKC*) and 7 out of 16 patients referred to our Clinical Unit in-season, showing signs and symptoms of disease (*active VKC*). The Total Symptom Score (TSyS) and the Total Sign Score (TSS) were, respectively, 7.33 ± 4.51 and 8.67 ± 3.05 (±SD). The mean overall scores were 0-1 in quiescent and 3-4 in active groups. A 1.27-fold increase of total protein concentration was quantified in quiescent samples while a 2.83-fold increase was detected in active VKC samples (*p* < 0.05, as compared to controls). Although a trend toward a decrease in total protein amount was detected in tears from the left eye, with respect to right one (*p* > 0.05), no significant intragroup changes were observed between active and quiescent total protein amounts. A representative total protein sketch is depicted in Figure 1, showing normalized VKC and control tear extracts resolved in a SDS-PAGE.

**Figure 2:**
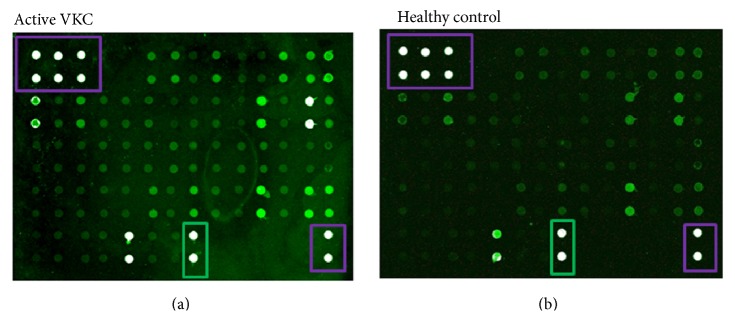
Overview of the protein array. Equal protein amounts were loaded for each subarray and the presence of an equal number of VKC: control samples were guaranteed for each array-chip (14 subarrays). (a), (b) Representative active VKC (a) and control (b) arrays, as provided by the GenePix scanner (with no color adjustment). White spots framed violet are positive controls, and black spots are negative referring controls and black spots framed green are albumin specific signals.

To recognize VKC proteins of prognostic value in a whole array of potential candidates, both quiescent and active VKC tears were subject to a chip-based protein array evaluation and appropriate statistical analysis. Proteins were selected from a literature search and antibodies were thereafter assembled. Both quiescent and active VKC tear samples were hybridized on chip-based arrays followed by a case-control statistical analysis. As shown in Figures 2(a)-2(b), an overall increased fluorescence was observed in VKC arrays (a), although few spots were also positive in controls (b). Protein expression profiles were similar in tears from left and right eyes. The whole array map is displayed in Table 2.

**Figure 3:**
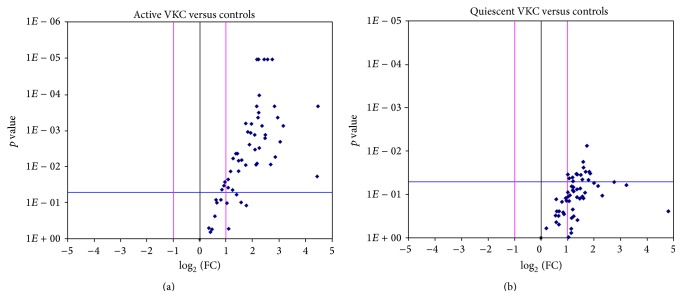
Plot graphs of the tear expression profile in active and quiescent VKC. Fold changes (Log_2_ (FC); *x*-axis) are ranked in Volcano plots according to the statistical significance (*p* values as negative Log_10_; *y*-axis). For each marker, FC between case and control values were calculated from mean of Fluorescent Intensity values provided by the software, as described in M&M. The two-sided unpaired *t*-test comparisons of active (a) and quiescent (b) samples were carried out versus controls. Both ±2 FC and *p* ≤ 0.05 were used as initial cut-offs. Red lines indicate differences of ±1 FC (log_2_) and blue line shows the initial significance level. Those candidates, having ≥2 FC and *p* ≤ 0.05 initial cut-offs, are localized in the upper left quadrant.

From this active VKC versus control comparison, 4 out of 16 candidates showed a ≥ 4.00-fold (*p* < 0.0001) differential expression (NT4, TACE, and TNF*α* converting enzyme allowing the cleavage of soluble form of TNF*α* receptor and Macrophage Inflammatory Protein (MIP) 1*α* and 3*α*); 16 out of 60 candidates had a ≥ 3.00-fold (*p* < 0.005) differential expression (cytokines IL1*β*, IL2, IL8, IL9, IL15, IL17, and IL21, growth factors NT4, BDNF, *β*FGF, and SCF, adhesion molecules TACE, MIP1*α*, MIP3*α*, and ICAM2/3, and soluble receptors sTNFRI/II) and 8 out of 16 candidates showed a ≥ 3.00-fold (*p* < 0.01) differential expression (IL4/5, IL12p40, IL16, IL17, IL18, and IL33/34). With respect to the expression of enzymes involved in the ECM metabolism, the MMP1 (5.81-fold with *p* < 0.0001) and MMP13 (5.36-fold; *p* < 0.0001), the MMP2 (3.49-fold; *p* < 0.005), and the MMP7 (6.35-fold; *p* < 0.01) were highly increased in active VKC tears, with respect to controls. While TIMP1/2 tissue inhibitors were not increased in active subgroup, the tissue inhibitor TIMP4 showed a significant increase in active VKC tear samples (4.44-fold; *p* < 0.01), as compared to controls. The Volcano plot underlying the complete protein expression in active VKC tears is shown in Figure 3(a).

From the quiescent VKC versus control comparison, some candidates were also increased in quiescent VKC tears (IL1*β*, IL15, IL21, *β*NGF, NT4, BDNF, *β*FGF, SCF, MMP1/2, Eotaxin2, TACE, MIP1*δ*, MIP3*α*, NCAM1, and ICAM2; ≥ 2.00-fold and *p* < 0.05, versus controls). MMP1 and MMP2 were found slightly increased in quiescent VKC tears (resp., 2.90- and 2.06-fold; *p* < 0.05). As above, a 3.94-fold increase was detected for TIMP4, although slightly significant (*p* = 0.051). Finally, IL33 expression was particularly high in both active (7.18-fold; *p* < 0.05) and quiescent (4.89-fold; *p* > 0.05) VKC tears, with respect to controls. The Volcano plot displaying the complete protein expression in quiescent VKC tears is shown, respectively, in Figure 3(b).

**Figure 4:**
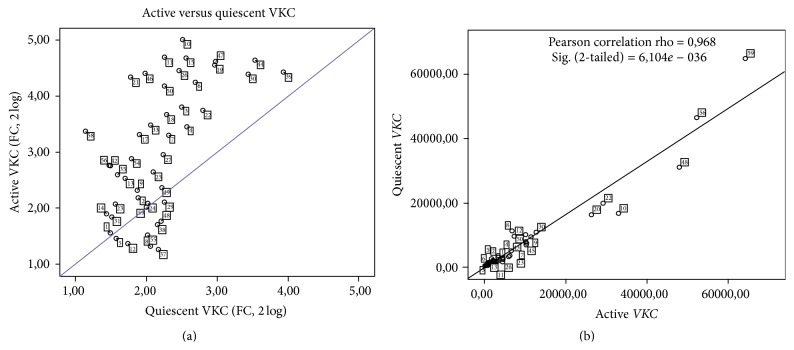
Comparison between quiescent and active subgroups. The scatter plot in (a) shows the candidate fold changes between quiescent and active tears, as calculated from mean of FI values provided by the software (see M&M). Those candidates, having ≥2 FC and *p* ≤ 0.05 initial cut-offs, are localized in the lower left quadrant. Correlation between quiescent and active biomarkers in VKC tears is shown in MFI values (b). The Pearson correlation analysis is reported in the panel. Note the close association of quiescent and active VKC candidates in the lower left region of the slope.

**Figure 5:**
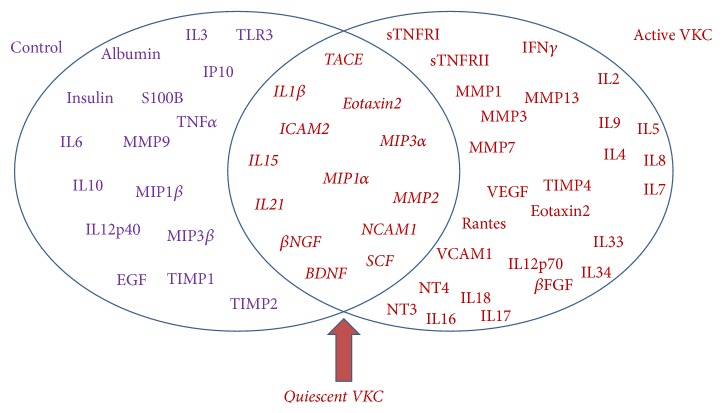
Venn diagram of predicted candidate biomarkers. Venn diagram showing the partial overlap between quiescent and active VKC groups. As predicted by this experimental approach, overlapping biomarkers are highlighted by the red arrow. At least in this study, all candidates showed at least ≥2-fold differences and a *p* value ≤ 0.05 or 0.00085, according to the Bonferroni correction.

The whole distribution of quiescent/active candidate fold changes in tear is shown in Figure 4(a) (Log_2_ expression). The scatter plot indicates that the majority of factors common to both VKC states are displayed in the lower region (low fold expression). Those factors highly expressed are mainly of active tear root (see upper quadrant in Figure 4(a)). The linear regression highlights the correlation between active and quiescent VKC tears (Pearson correlation rho = 0.968, *p* = 6.104*e* − 036; Figure 4(b)). As summarized in Figure 5, 16 out of 60 candidates overlap active and quiescent VKC states and include the following: IL1*β*, IL15, IL21 (inflammatory cytokines), *β*NGF, NT4, BDNF, SCF (growth factors), MMP1, MMP2 (tissue proteases) and Eotaxin2, TACE, MIP1*α*, MIP3*α*, NCAM1, and ICAM2 (chemokines and adhesion molecules). A further analysis of quiescent versus active VKC indicated that three out of those 16 candidates (NT4, *β*FGF, and MMP1; see bold font in Table 3) were significantly expressed in the active form with respect to the quiescent group (*p* < 0.05). Albeit not significant, MIP1*β* was extremely high in both active and quiescent specimens (resp., 32.86-fold and 27.61-fold; both *p* > 0.05 versus controls).

## 4. Discussion

Currently in force for a wide-range biomolecular investigation of disease-linked profiles, the microarray platform represents an excellent high-throughput technology that facilitates the simultaneous detection of more than a few “biomarker or gene/oligo/protein candidates” [14, 15, 25]. By using the protein-based approach set up in fluorescence, we addressed the question as to whether some protein candidates (prospective indicators of inflammation and/or tissue remodeling) might be expressed in both active and quiescent VKC tears, representing pointer of disease activity and useful prognostic factors in the clinical practice. As discussed below, we confirmed some protein candidates and identified new ones in active VKC and recognized some common to both quiescent and active forms. These acute and quiescent overlapping candidates are IL1*β*, IL15, IL21, *β*NGF, BDNF, SCF, MMP2, Eotaxin2, TACE, MIP1*α*, MIP3*α*, NCAM1, and ICAM2, as shown in the Venn diagram in Figure 5 (pointed by red arrow).

Firstly, the widespread protein expression in active VKC with respect to control tears is in line with the inflammatory and tissue remodeling process occurring at the ocular surface. Cytokines, chemokines, and adhesion molecules, growth factors, and some soluble receptors play a major role in chronic inflammatory disorders, through a tidy regulation of cell influx leading to an exacerbation of the local inflammation and the promotion of corneal lesions [3]. The observation of the increased expression of IL1*β*, IL15, IL21, Eotaxin2, TACE, MIP1*α*, MIP3*α*, NCAM1, ICAM2, *β*NGF, NT4, BDNF, *β*FGF, SCF, MMP1, MMP2, and sTNFRI/II is in line with literature, as reported in previous studies carried out on VKC tears, ICs, and conjunctival biopsies [5–13]. Particularly, IL9 has been associated with seasonal-mediated allergic activation [26, 27]; IL7, IL15, and IL21 have been reported in chronic inflammatory and tissue remodeling process [27, 28]; IL17 has been detected only in VKC sera [27, 29]; IL18 has been implicated in Th1/Th2-response modulation [30], and IL16 has been correlated with IgE level expression [31]. The unchanged TNF*α*, IL6, and IL10 expression and slight IFN*γ* increase in active VKC might be consistent with the absence of corneal involvement and tissue remodeling, as provided by the VKC score [12, 27, 32]. The high expression of IL33 and IL34 in active VKC might be linked to the Th2-response and the IgE dependent activity, as well as to the release of Th2-derived cytokines through a stimulatory effect on mast cells, eosinophils, and basophils [33].

Although at a relatively low level, the common expression of some candidates in quiescent VKC tears would imply that these factors might provide long-lasting conditions suitable for disease reactivation and therefore might be proposed as a potential indicator of forthcoming reactivation. Of those cytokines significantly expressed in active VKC tears (IL3, IL7, IL9, IL15, IL16, IL17, IL18, IL21, and IL33/34), only IL1*β*, IL15, and IL21 were monitored in quiescent VKC. Overexpressed in active VKC, Eotaxin2 detection in quiescent tears might be indicative of a forthcoming eosinophil recruitment, according to* in vitro*/*in vivo* chemoattractant studies [34]. In addition, MIP1*α*, MIP3*α*, TACE, NCAM1, and ICAM2 might represent new potential candidates for their well-known cell homing effects [34–37]. It is noteworthy that MIP1*α*-MIP3*α* has been recently implicated in the epithelial cell activation as well as eosinophil, lymphocyte, and neutrophil tissue-homing and, more appropriately, MIP3*α* has been recently prospected as a serum prognostic factor at least for systemic inflammatory diseases [37]. NCAM1 and ICAM2 expression in quiescent VKC tears might be suggestive of a sustained local Th2-driven response as well as neutrophils, NK cell, eosinophil, and mast cell noiseless activity [10, 12, 34–37]. Finally, the common expression of TACE in both active and quiescent VKC tears might be indicative of a potential synthesis/release of IL8, a potent regulator of neutrophil activity [36].

A cross-talk between neuronal and nonneuronal cells has been described in VKC and a bidirectional (neuro)protective and/or anti-inflammatory cross-talk between immune and structural cells might be prospected under quiescent conditions [13, 38, 39]. While NGF, BDNF, NT3/4, SCF, VEGF, and TGF*β*1 changes in active VKC might be due to production by activated eosinophil, mast cell, and T cells [40, 41], the observation of NGF, NT4, BDNF, and *β*FGF expression in quiescent VKC is actually an open question [41, 42]. Some neuroprotective effects might be proposed for NGF, NT3/4, and BDNF overexpression in quiescent VKC tears [39]. On the contrary, SCF expression in quiescent VKC tears might be explained with the active VKC microenvironment characterized by chemotactic/survival/regulatory activities of mast cell and eosinophils [43].

Several tissue proteases/inhibitors (MMPs/TIMPs) trigger long-lasting ECM metabolism in active VKC, while playing homeostatic effects under normal states [1, 2]. The uncontrolled MMPs/TIMPs might significantly contribute to chronic-sustained extensive tissue remodeling and giant papillae formation, by means of collagen/basal membrane degradation and inflammatory cell transmigration, until the development of corneal erosions, as observed in severe VKC forms [15, 44]. Except for MMP1/MMP2/MMP13 previously documented in VKC, the selective MMP7/TIMP4 overexpression in active VKC and the common expression of MMP2 in both quiescent and active forms represent new attractive findings. Apparently not in line with previous studies, the slight MMP9 expression in active VKC is consistent with the unchanged TNF*α* values (a major MMP9 modulator) and might be supported by the absence of superficial corneal involvement or ulcers in this study population [44]. As reported, the main MMP9 activity occurs at the corneal basement and correlates with papillae development and corneal erosion in VKC [44]. The significant increase of the regulatory MMP7 appears to be of great interest. MMP7 might allow the activation of other MMPs and the accessibility of several neurotrophic/angiogenic factors (VEGF, TGF*β*1, and NGF) [15, 45]. With respect to NGF-MMP7 interaction, the proNGF cleavage has been recently associated with some neuroprotective activities [45].

Overall, VKC is a self-limiting eye disease characterized by a chronic inflammatory process (mostly seasonal driven) and overt ECM remodeling [1–6]. None of the current therapies available can really protect from VKC discomfort and corneal hurt, suggesting that any attempts to offset the recurrent/long-standing inflammation (disease/complications) or foresee the seasonal activation/exacerbation are welcome from ophthalmologists [16–18]. Since some tear proteins represent the output of cells populating the ocular surface (merely conjunctival epithelium/stroma) and since VKC prolongation is dependent on physical/biological stressors and microenvironment, the identification of some laboratory markers common to acute and quiescent VKC forms might represent a foremost target for developing alternative strategies to counteract VKC reactivation. Herein, a predictive VKC-tear protein profile might be hypothesized for IL1*β*, IL15, IL21, Eotaxin2, TACE, MIP1*α*, MIP3*α*, NCAM1, ICAM2, *β*NGF, NT4, BDNF, *β*FGF, SCF, MMP1, and MMP2, all quantified in acute VKC and detected also in quiescent VKC tears. Moreover the eye-flush tear sampling technique appears appropriate as it allows an easy collection and a good volume (diluted tears) for the chip-based array. The specificity of this way of sampling is provided by the observation that only a few of the significantly changed proteins were identified.

Understanding the potential candidates might be helpful for prognostic purposes as well as for developing appropriate strategies to counteract VKC either outside or during the season, and with respect to other VKC-associated conditions (i.e., dry eye).

## Figures and Tables

**Table 1:** Study population: overall description of some demographic and biochemical data of quiescent and active subgroups.

Patient group	VKC	
Number (M/F)	16 (15/1)	
Therapy (topical/systemic)	none	

Patient subgroups	Quiescent	Active

Number	9	7
Seasonal gap	outside	inside
Mean overall score	0-1	3-4
Tear protein content (mean ± SD)^§^	3.67 ± 0.84	8.19 ± 1.56

^§^Tears were sampled with the eye-flush technique and total proteins were measured according to the A280 Nanodrop program (see M&M). Total protein concentration in control tear samples: 2.89 ± 0.56. Total protein values are expressed in *μ*g/*μ*L.

**Table 2:** The complete list of 60 capture antibodies is reported, as spotted onto each subarray (see Figure 2).

0	A	B	C	D	E	F	G	H	I	J	K	L	M	N
1	POS 1	POS 2	POS 3	NEG	NEG	TNF-alpha	IFN-gamma	IL-1 beta	IL-2	IL-3	IL-4	IL-5	IL-6	IL-7
2	POS 1	POS 2	POS 3	NEG	NEG	TNF-alpha	IFN-gamma	IL-1 beta	IL-2	IL-3	IL-4	IL-5	IL-6	IL-7
3	IL-8	IL-9	IL-10	IL-12p40	IL-12p70	IL-17	IL-18	MMP-1	MMP-2	MMP-7	MMP-9	MMP-13	TIMP-1	TIMP-2
4	IL-8	IL-9	IL-10	IL-12p40	IL-12p70	IL-17	IL-18	MMP-1	MMP-2	MMP-7	MMP-9	MMP-13	TIMP-1	TIMP-2
5	TIMP-4	Eotaxin	Eotaxin2	RANTES	TACE	MIP-1alpha	MIP-1beta	MIP-1delta	MIP-3alpha	MIP-3beta	TLR2	IL-33	IL-34	Insulin
6	TIMP-4	Eotaxin	Eotaxin2	RANTES	TACE	MIP-1alpha	MIP-1beta	MIP-1delta	MIP-3alpha	MIP-3beta	TLR2	IL-33	IL-34	Insulin
7	VCAM-1	NCAM-1	ICAM-1	ICAM-2	ICAM-3	IL-15	IL-16	IL-21	sTNF RI	sTNF RII	IP-10	b-NGF	VEGF	TGF-beta1
8	VCAM-1	NCAM-1	ICAM-1	ICAM-2	ICAM-3	IL-15	IL-16	IL-21	sTNF RI	sTNF RII	IP-10	b-NGF	VEGF	TGF-beta1
9	NT-3	NT-4	BDNF	bFGF	EGF	SCF	S100B	Albumin	NEG	NEG	NEG	NEG	NEG	POS-2
10	NT-3	NT-4	BDNF	bFGF	EGF	SCF	S100B	Albumin	NEG	NEG	NEG	NEG	NEG	POS-2

**Table 3:** Summary of the protein profile expression in all subgroups. The 16 differentially expressed proteins (candidate biomarkers) are functionally grouped in specific clusters: cytokines (Th1-Th2-Th9-Th17 subtypes), growth factors (neurotrophins and fibrogenic/angiogenic factors), chemokines/adhesion molecules, tissue proteases (specific ECM enzymes/inhibitors), and other molecules (soluble receptors and referring proteins). Column 2 provides the significance (Sig.) for active and quiescent biomarkers, as obtained with respect to controls (^*∗*^ *p* < 0.05; ^*∗∗*^ *p* < 0.01; ^*∗∗∗*^ *p* < 0.005; ^*∗∗∗∗*^ *p* < 0.0001; two-sided unpaired *t*-test analysis). The related quiescent: active comparisons (fold changes (FC)), *p* values and significances are shown. Bold font indicates candidates common to quiescent and active VKC (*p* > 0.05). The last column indicates each candidate incthe related VKC literature and the § symbol highlights only those papers concerning VKC tissues.

Clusters Candidate biomarkers	Sig., cases versus controls		Active versus quiescent			Literature
	Quiescent	Active	FC	*p* value	Sig.	Ref
Cytokines						
IL1*β*	**∗**	**∗** **∗** **∗**	***1.52***	***0.2326***		**[6]**
IL15	**∗**	**∗** **∗** **∗**	***1.83***	***0.0628***		
IL21	**∗**	**∗** **∗** **∗**	***1.54***	***0.1714***		
Growth factors						
*β*NGF	**∗**	**∗** **∗**	***1.34***	***0.3885***		**[13, 40]** ^§^
NT4	*∗*	*∗∗∗∗*	*1.81*	*0.0410*	*∗*	
BDNF	**∗**	**∗** **∗** **∗**	***1.33***	***0.3580***		**[41]**
*β*FGF	*∗*	*∗∗∗*	*2.24*	*0.0286*	*∗*	[41]
SCF	**∗**	**∗** **∗** **∗**	***1.28***	***0.4784***		**[41]**
Tissue proteases						
MMP1	*∗*	*∗∗∗∗*	*2.01*	*0.0417*	*∗*	[41]
MMP2	**∗**	**∗** **∗** **∗**	***1.69***	***0.0764***		**[41]**
Eotaxin2	**∗**	**∗**	***3.19***	***0.1132***		**[6, 34, 42]**
TACE	**∗**	**∗** **∗** **∗** **∗**	***1.95***	***0.0554***		
MIP1*α*	**∗**	**∗** **∗**	***1.32***	***0.4775***		
MIP3*α*	**∗**	**∗** **∗** **∗** **∗**	***1.55***	***0.1446***		
NCAM1	**∗**	**∗** **∗**	***1.85***	***0.0789***		
ICAM2	**∗** **∗**	**∗** **∗** **∗**	***2.34***	***0.0226***		**[34]**

Two-sided unpaired *t*-test analysis (fold changes (FC), *p* values, and case/control significance (Sig.; ^*∗*^ *p* < 0.05; ^*∗∗*^ *p* < 0.01; ^*∗∗∗*^ *p* < 0.005; ^*∗∗∗∗*^ *p* < 0.0001)) (see M&M).
